# Construction of a novel coarse grain model for simulations of HIV capsid assembly to capture the backbone structure and inter-domain motions in solution

**DOI:** 10.1016/j.dib.2015.09.042

**Published:** 2015-10-09

**Authors:** Xin Qiao, Jaekyun Jeon, Jeff Weber, Fangqiang Zhu, Bo Chen

**Affiliations:** a Department of Physics, University of Central Florida, 4000 Central Florida Blvd, Orlando, FL 32816, USA; b Department of Physics, Indiana University – Purdue University Indianapolis, IN, USA

**Keywords:** HIV, Capsid assembly, Coarse grain simulations

## Abstract

We show the construction of a novel coarse grain model for simulations of HIV capsid assembly based on four structural models of HIV capsid proteins: isolated hexamer 3H47.pdb, tubular assembly 3J34.pdb, isolated pentamer 3P05.pdb and C-terminus dimer 2KOD.pdb. The data demonstrates the derivation of inter-domain motions from all atom Molecular Dynamics simulations and comparison with the motions derived from the analysis of solution NMR results defined in 2M8L.pdb. Snapshots from a representative Monte Carlo simulation with 128 dimeric subunit proteins based on 3J34.pdb are shown in addition to the quantitative analysis of its assembly pathway. Movies of the assembly process are compiled with snapshots of representative simulations of four structural models. The methods and data in this article were utilized in Qiao et al. (in press) [1] to probe the mechanism of polymorphism and curvature control of HIV capsid assembly.

**Specifications Table**

**Table:** 

Subject area	*Biophysics*
More specific subject area	*Coarse grain simulations of viral capsid assembly*
Type of data	*Figures and description of simulation procedures*
How data was acquired	*Microscope, survey, SEM, NMR, mass spectroscopy, etc. If an instrument was used, please provide the model and make of the instrument*
Data format	*Raw and analyzed*
Experimental factors	*Data is acquired with all atom simulation by NAMD. Assembly simulations are performed by coarse grain models and analyzed by self-developed TCL scripts in VMD. Movies are made in VMD*
Experimental features	*Simulations of HIV assembly with novel coarse grain models*
Data source location	*University of Central Florida, Orlando FL 32828, USA*
Data accessibility	*On the computer of corresponding author*

***Value of the data***

1. We show a novel coarse grain model that accurately represents the subtle variations of backbone structure of rigid segments of the protein.
2. We show a novel strategy to represent the inter-domain motions in coarse grain simulations by a static ensemble of subunits with variable domain orientations derived from analysis of all atom Molecular Dynamics simulations, provided that the structure within each domain remains unchanged.
3. The method and approach used in this work can be generalized for other protein assemblies with similar characteristics.

## 1 Data

Polymorphism and the continuous variation of curvatures are two peculiar features of HIV capsids [2]. A HIV capsid protein consists of two independently structured domains, the N-terminus domain (NTD) and C-terminus domain (CTD), linked by a short flexible inter-domain linker [3]. HIV capsid proteins dimerize via helix 9 at its CTD in solution, and can form polymorphic assemblies *in vitro* [3]. Various structural models of such assemblies were determined, stabilized by three intermolecular contacts: NTD–NTD, NTD–CTD and trimeric interfaces [3]. We demonstrate the construction of a novel coarse grain (CG) model that captures the subtle variations of backbone structure of HIV capsid proteins and a strategy to account for protein dynamics with a static ensemble of subunits in conformations derived from all atom Molecular Dynamics (MD) simulations. Simulations using this novel CG model and strategy demonstrate that the variations of inter-domain motions controls the curvature of the assembly and causes the polymorphism, as show in Ref. [1]. In this article, we focus on the illustration of CG model conversion from the template pdb files and extraction of inter-domain motions from all atom MD simulation and solution NMR data.

**Fig. 1:**
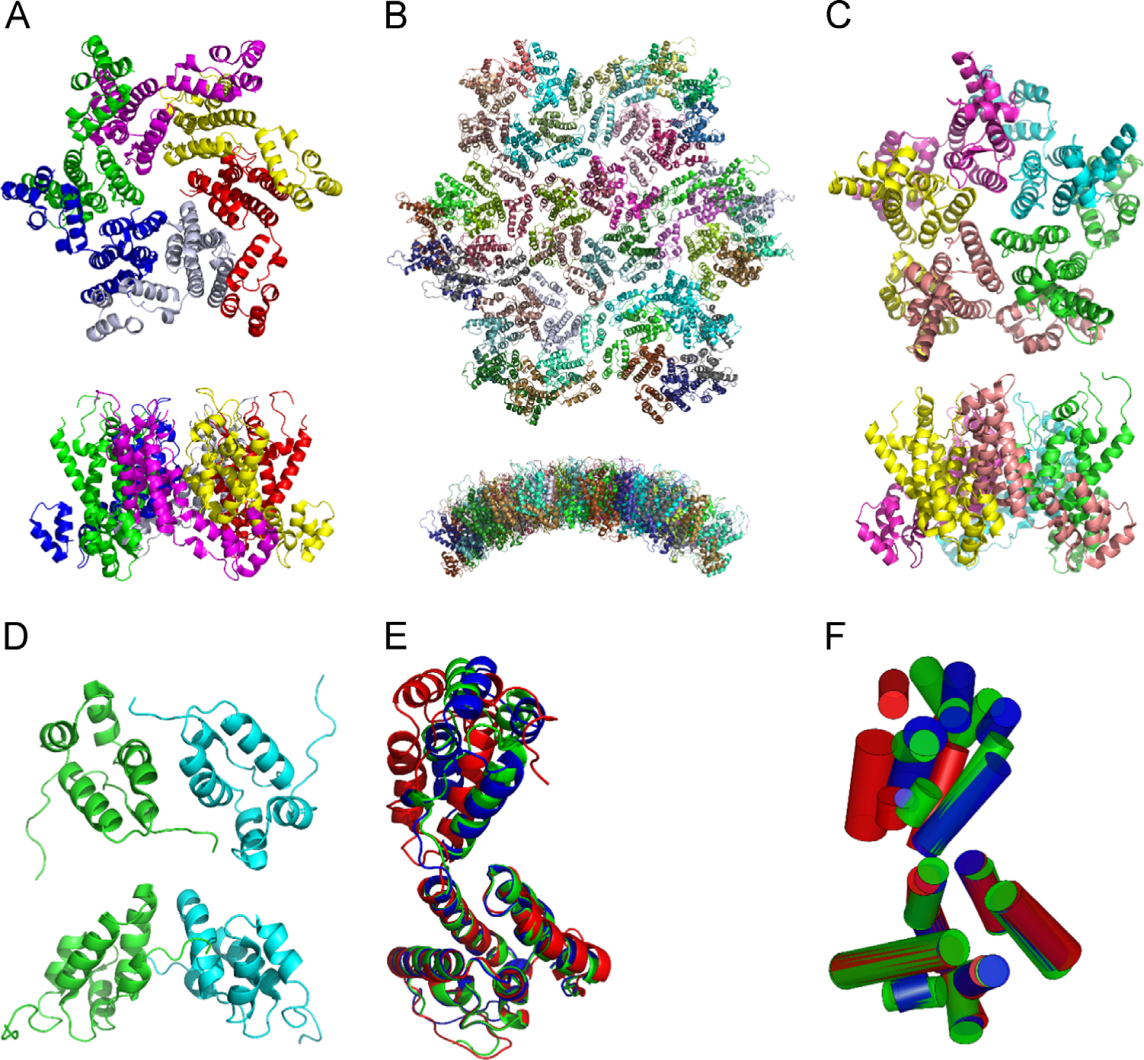
Structural template pdb files used for CG model construction and their comparison. (A) Isolated hexamer 3H47.pdb. (B) Tubular assembly 3J34.pdb. (C). Isolated pentamer 3P05. (D) CTD dimer 2KOD.pdb. In (A–D), a top view and side view of each template are plotted and individual monomers in each template are plotted in distinct colors. (E, F) Comparison of monomers from 3H47.pdb (green), 3J34.pdb (red), and 3P05 (blue). They are aligned using their NTDs as references in (E) on the left, compared to their CG models in (F) on the right. Their NTDs exhibit a good alignment. Helices/Cylinders in CTDs show large deviations due to the different overall orientation of two domains. The RMSD to align 3H47 and 3P05 is 0.839 Å, and the RMSD to align 3J34 and 3P05 is 1.780 Å.

Fig. 1 illustrates the structural differences of the four experimental structural models of HIV capsid proteins utilized as templates for our novel coarse grain model: the isolated hexamer 3H47.pdb, tubular assembly 3J34.pdb, isolated pentamer 3P05.pdb and C-terminus dimer 2KOD.pdb. We show that our CG model uses cylinders to capture the subtle variations of backbone structures in these templates.

**Fig. 2:**
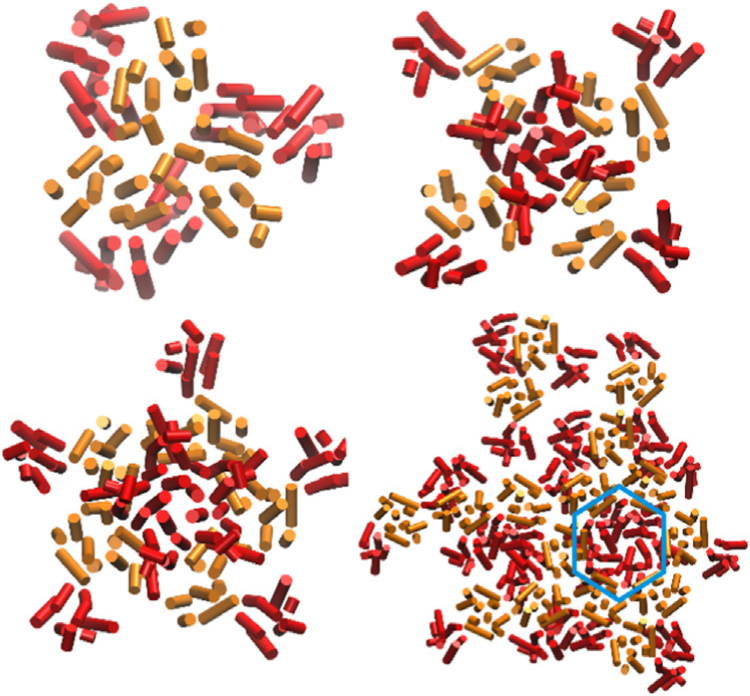
Important oligomer intermediates observed in simulations. Top left: a trimer of dimeric subunits. Top right: a tetramer of dimeric subunits. Bottom left: a pentamer of dimeric subunits. Bottom right: formation of hexameric lattice where a hexamer is highlighted in the center.

Fig. 2 lists the four critical assembly intermediates identified in our simulations, which can be seen in the movies (Movies 1 to 5) compiled with snapshots of representative simulations of four structural models.

**Fig. 3:**
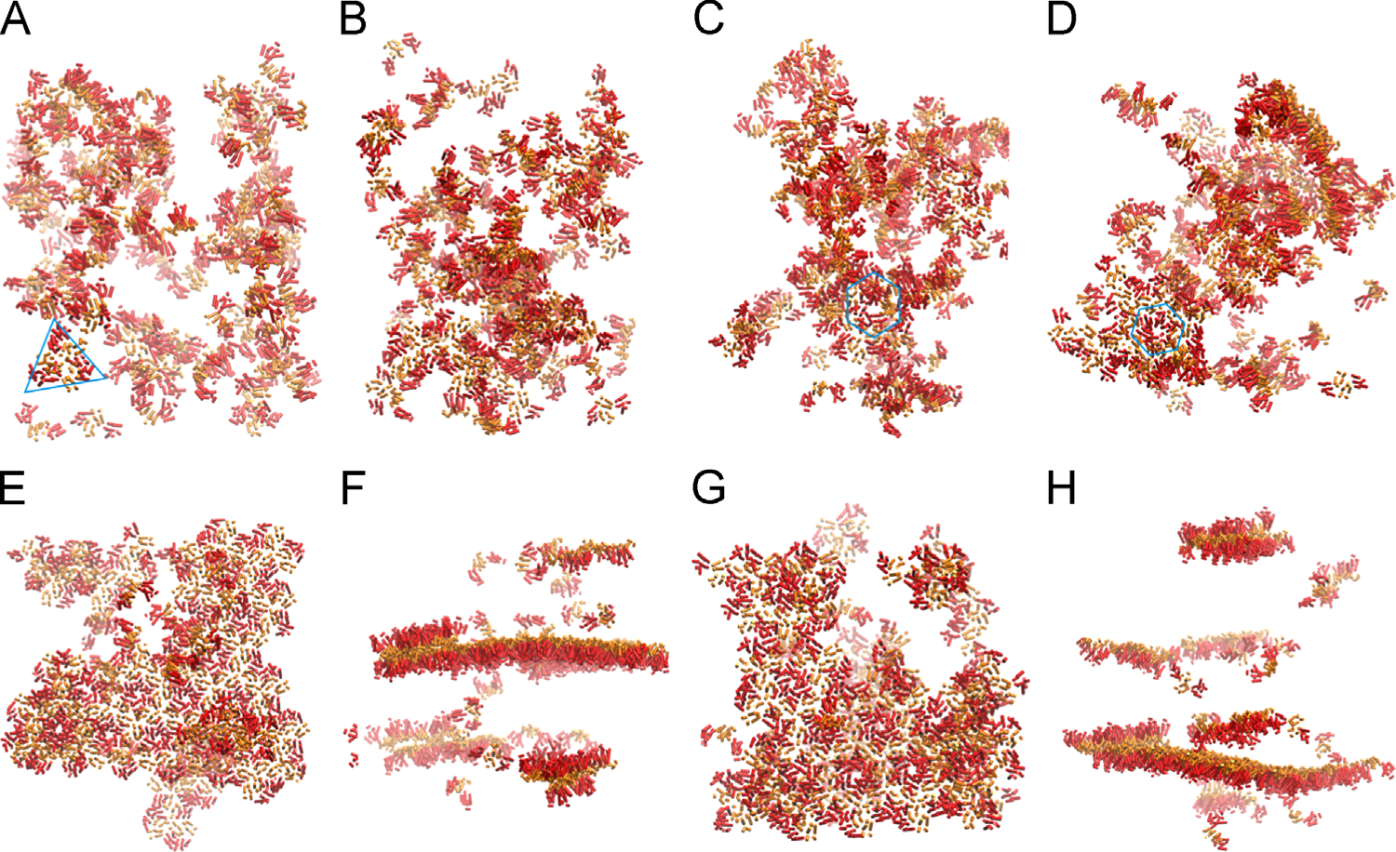
Simulations of 128 dimeric subunits based on tubular assembly model 3J34.pdb. (A) Snapshot at step 200 million. Various oligomers at the early stage of assembly. A trimers is highlighted by a blue triangle. (B). Snapshot at step 1200 million. Trimers dominate the system. (C) Snapshot at step 3400 million, just before the assembly of a hexamer, highlighted by the blue hexamer. (D) Snapshot at step 3760 million, a lattice of multiple hexamers formed around the hexamer. (E, F) Top and side views of the system at step 15,134 million. A large curved hexameric lattice and a couple of small hexamer patches are present. (G, H) Top and side views of the system at step 17,620 million. The curvature of the large hexameric lattice differs from that in (F). Double-layered lattices could be seen in both (F, H).

Fig. 3 illustrates the assembly of a system comprised of 128 dimeric subunits based on 3J34.pdb. Representative trimer and hexamers are highlighted in Fig. 3A, C and D. The quantitative analysis of the assembly pathway of the system is shown in Fig. 7.

**Fig. 4:**
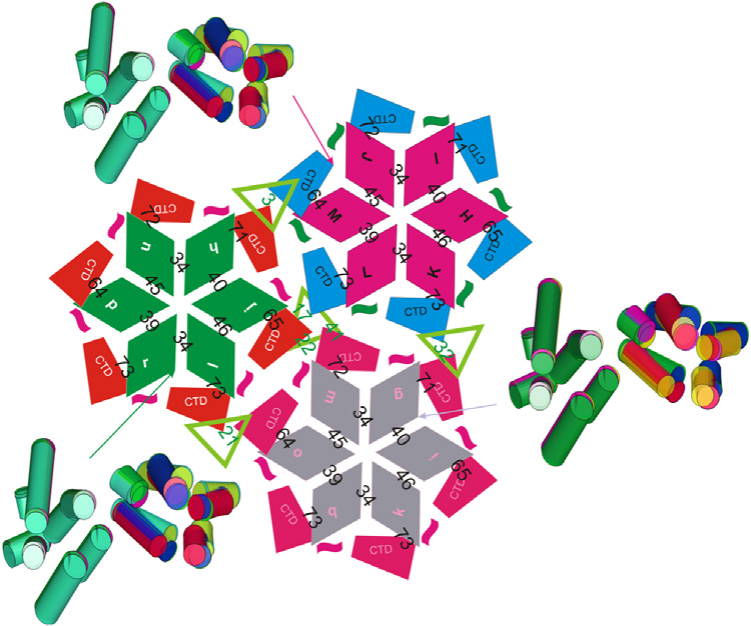
The variations of subunit structure and interactions in tubular assembly 3J34.pdb. In this schematic assembly figure at the center, NTDs are represented by diamonds, with the letters corresponding to the molecular segment label in 3J34.pdb [14]. CTDs are represented by trapezoids, with the short ribbons indicating the connection to its corresponding NTDs. Trimeric interfaces are highlighted by the green triangles between CTDs. The numbers between NTDs are the contact angles between helices 2 and 3 at NTD–NTD interfaces. The numbers between CTDs and NTDs denote the contact angles between helices 4 and 10 at NTD–CTD interfaces. The numbers in green triangles are the contact angles between helices at trimeric interfaces. The CG models for six monomers in each hexamers are plotted on the side in different colors, aligned to their NTDs.

Fig. 4 shows the variation of contact angles of neighboring subunits in 3J34.pdb between NTD–NTD, NTD–CTD, and CTD–CTD interfaces.

**Fig. 5:**
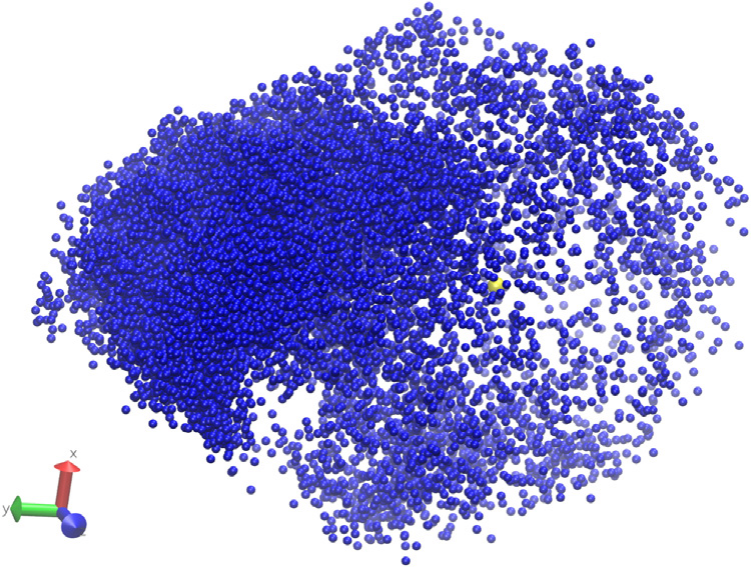
Rotations to realign NTDs along the trajectory of 303 ns MD simulations of a dimer based on 3J34.pdb. The yellow sphere represent the center of rotation, which is the COM of the dimer. Each small blue sphere represents a rotation necessary to align NTD at a later time point to the original NTD at *t*=0 in the 303 ns MD simulations based on chains A and f in 3J34.pdb: The distance between the blue and yellow spheres represents the magnitude of the rotation angle, and the rotation axis is represented by a line connecting the yellow and the corresponding blue sphere.

Fig. 5 shows the rotation necessary to align the NTDs of the dimer at subsequent time points to *t*=0 along the trajectory of the 303 ns all atom MD simulations of a dimer based on chains A and f in 3J34.pdb.

**Fig. 6:**
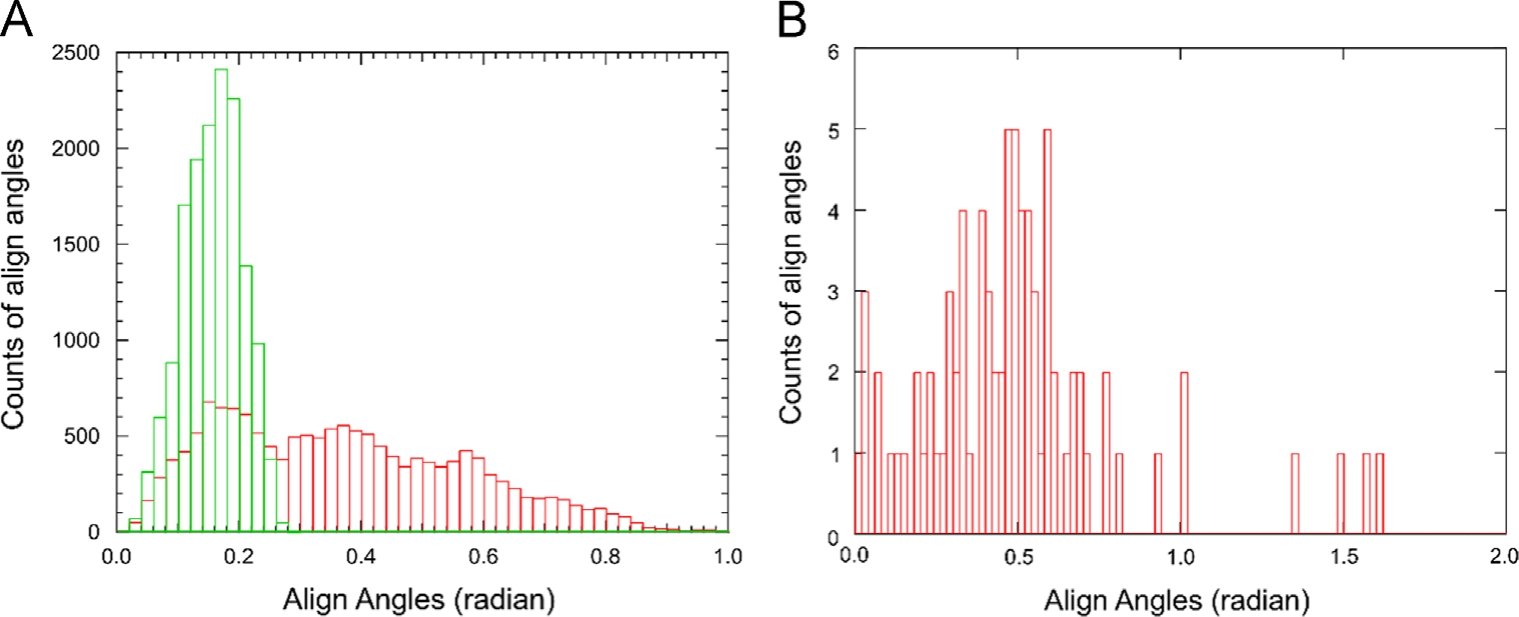
Flexibility of NTDs to CTDs orientation in a dimeric HIV capsid protein. The flexibility of NTD and CTD is measured by the angles to align each domain at a later time point to their counterpart in the trajectory of all-all MD simulation, or different frames in solution NMR structural model 2M8L.pdb. Results from analysis applied to the 303 ns MD simulations based on chains A and f in 3J34.pdb [6], shown in (A), and analysis applied to realign 3J34.pdb branch g to each of the 100 dimers in solution NMR determined ensembles 2M8L.pdb, shown in (B). The angle for NTD is colored in red, and for CTD colored in green. All CTDs in 2M8L are identical, so no statistics shown in B for CTDs.

Fig. 6 shows the flexibility of the NTDs and CTDs in a dimeric HIV capsid protein measured by the angles to align the domains along the trajectory of the 303 ns all atom MD simulations of a dimer based on chains A and f in 3J34.pdb (in A), compared to that measured by solution NMR (in B).

**Fig. 7:**
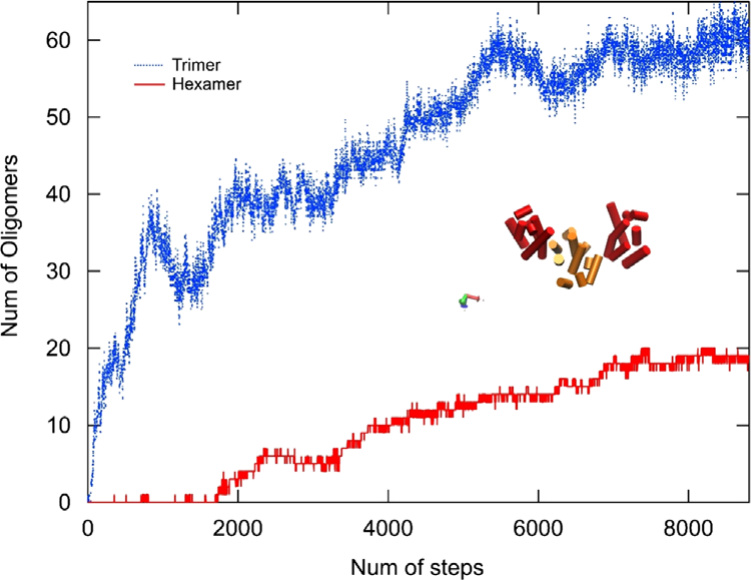
Assembly pathway of an individual simulation based on 3J34.pdb. The system consists of 128 identical subunits. As shown by the evolution of hexamers, occasions of transient assembly of hexamers are present at the early stage of the assembly, but they disappear almost instantaneously.

Fig. 7 shows the quantitative analysis of the assembly pathway of a representative simulations (snapshots of the assembly shown in Fig. 3) with 128 dimeric subunits based on 3J34.pdb.

Detailed view of the evolution of simulations based on different structural templates are shown by Movies 1–5, compiled with snapshots taken from respective simulations.

## 2 Experimental design, materials and methods

### 2.1 Procedures to extract NTD and CTD orientations from MD simulation

Using NAMD [4], [5], a trajectory of 303 ns all atom MD simulation was performed on a dimer based on chains f and A in 3J34.pdb [6] with step size of 2 fs. The CTDs of the dimer at each subsequent time point were realigned with the CTDs of the dimer at *t*=0. Then, another rotation was applied to align their NTDs. The angle and rotation axis to align the dimer in each subsequent time point along the MD trajectory was obtained by retrieving the 4×4 rotation matrix to complete the realignment function. The center of rotation is the Center of Mass (COM) of the dimer. Altogether 151,501 set of angles and directional vectors were obtained from this analysis. The angles derived from this analysis is shown in Fig. 5. This analysis was also applied to analyze the solution NMR structure ensemble of HIV capsid protein 2M8L consisting of 100 dimers [7], shown in Fig. 6.

### 2.2 Set up a system consistent with variable NTD and CTD orientations within dimeric subunits in MD simulation

First, generate a random integer between 1 and 151,501. Then use this integer as the index to choose the set of angle and axis of rotation from the analysis of MD simulation as described above. Apply the corresponding rotations to the CTDS and NTDs.

### 2.3 Details for all atom Molecular Dynamics simulation of a HIV capsid dimer

The simulation was performed using the CHARMM22 force field with the CMAP correction [8], [9], [10] under the periodic boundary conditions and the NVT ensemble. All bond lengths involving hydrogen atoms were constrained using the SHAKE [11] algorithm. We adopted a cutoff distance of 12 Å for nonbonded interactions, with a smooth switching function taking effect at 10 Å. Full electrostatics was calculated every 4 fs using the particle-mesh Ewald method [12]. Temperature was maintained at 330 K by Langevin dynamics with a damping coefficient of 1 ps^−1^.

CG model construction and setup of simulations are explained in detail in Ref. [1]. Simulations were performed at UCF Stokes ARCC and Edison system of National Energy Research Scientific Computing Center (NERSC). Figures and movies are created using Matlab (The MathWorks, Natick, MA), Pymol (Schrödinger, LLC.), and VMD with self-developed TCL scripts [13].
