# Supplementary material for: Construction of a novel coarse grain model for simulations of HIV capsid assembly to capture the backbone structure and inter-domain motions in solution
Source: Data Brief. 2015 Oct 9;5:506–12. doi: 10.1016/j.dib.2015.09.042 (PMC4631880; doi:10.1016/j.dib.2015.09.042)
Supplement: Supplementary file 1 — Supplementary material [file mmc1.doc]

**Movie list:**

**Movie 1.** Assembly of flat hexamer lattice of HIV capsid protein. The system consists of 128 identical dimeric subunits based on hexamer template 3H47.pdb, with dimer interface modeled after 2KOD.pdb. The movie is constructed for the system shown in Fig. 1 in the manuscript.

<https://youtu.be/q1Y_bdktfQA>

**Movie 2.** Assembly of curved hexameric lattices. The system consists of 128 identical dimeric subunits based on tubular assembly 3J34.pdb.

<https://youtu.be/SRHB8YZtdB4>

**Movie 3.** Impact of protein dynamics on assembly. The system consists of 128 dimeric subunits with variable NTD-CTD orientation. The distribution of domain orientation is derived from 303 ns MD simulation based on a dimer (segment A and f) in tubular assembly 3J34.pdb. <https://youtu.be/ad27fQVdzeU>

**Movie 4.** Assembly of highly curved structure by HIV capsid proteins. The system consist of 128 identical dimeric subunits based on the pentameric template 3P05.pdb and dimerization interface based on 2KOD.pdb. The movie is constructed for the system shown in Fig. 5.

<https://youtu.be/w-YdvuaAEjc>

**Movie 5.** Impact of subunit interaction on assembly. The system consists of 128 identical subunits whose structure are based on hexamer template 3H47.pdb, but interactions modeled after the pentamer template 3P05.

<https://youtu.be/IkghhArBo3U>
