# Supplementary figures and images for: Construction of a novel coarse grain model for simulations of HIV capsid assembly to capture the backbone structure and inter-domain motions in solution
Source: Data Brief. 2015 Oct 9;5:506–12. doi: 10.1016/j.dib.2015.09.042 (PMC4631880; doi:10.1016/j.dib.2015.09.042)

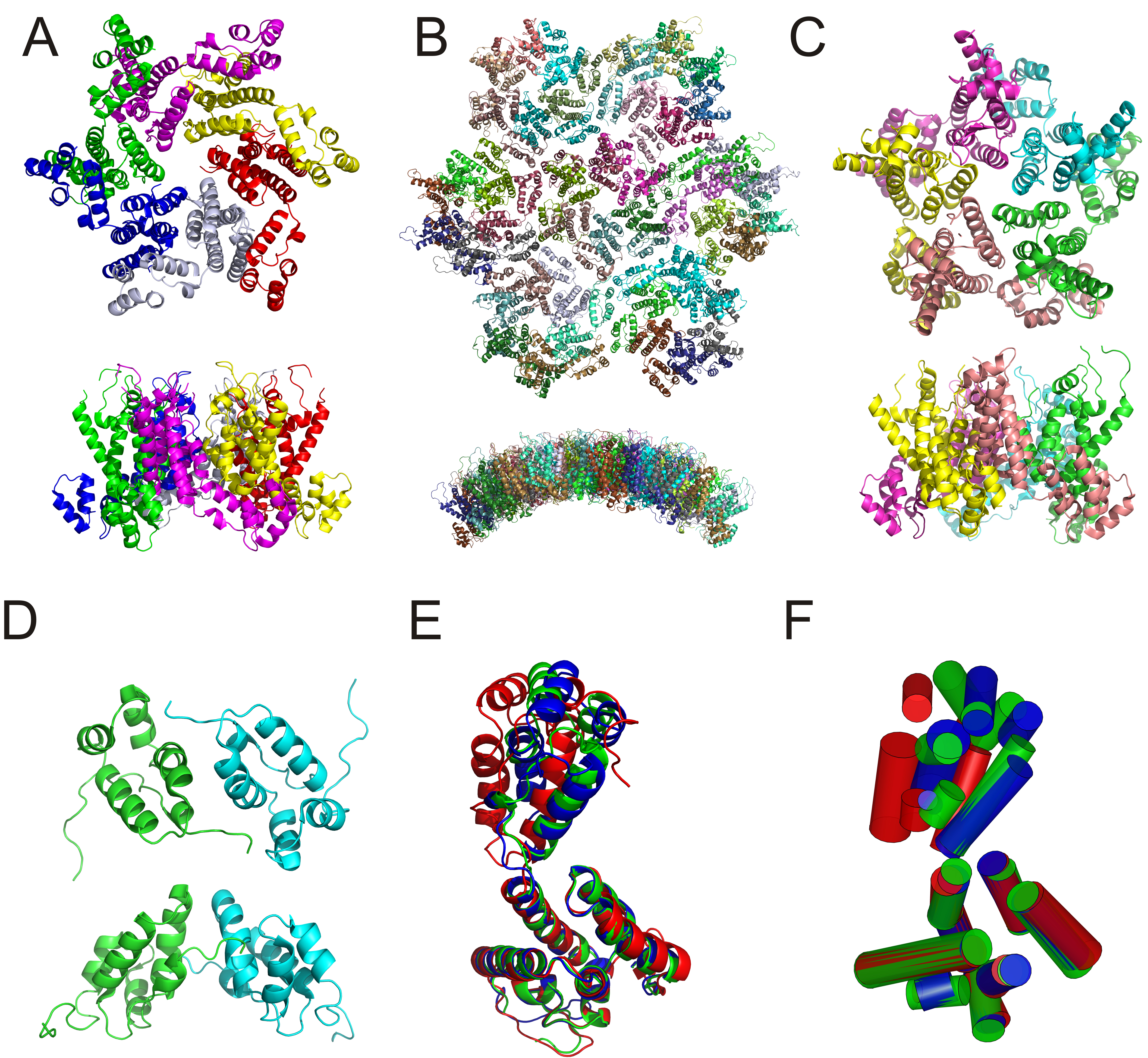

Supplement: Supplementary file 3 — Supplementary material [file mmc3.zip › FigureS1.tif]
